# Supplementary material for: Chromothripsis during telomere crisis is independent of NHEJ, and consistent with a replicative origin
Source: Genome Res. 2019 May;29(5):737–49. doi: 10.1101/gr.240705.118 (PMC6499312; doi:10.1101/gr.240705.118)
Supplement: Supplemental Material [file supp_gr.240705.118_Supplemental_file_1.zip › contigs/annotated_contigs/DB83/contig.2.DB83_length_620_mean_cov_10.0435483871.docx]

**DB83_length_620_mean_cov_10.0435483871**

```
CTTATTTCTCTCTGCCTGGTTTTCTCCCGAGTCTGGAAACTACCTGTGAGTATCCTTAAATTATGGCAGTATGGTTATTTGCAAAGGTG
 >chr2:10341087-10341411 - E=3e-177
CAATACAAGTCTGTTTTCTTTCTTTCTTTCTTTCTTTTTTTTTTTTGAGATGGAATCTTGCTCTGTTCCCCAGGCTGGAGAGCAGTGGT

GCAATCTCGGCTCACTGCAACCTCCACCTCCCAGGTTCAAGCAATTCTCCCTGCCTCAGCCTCCCGAGTAGCTGGGATTACAGGCATGC

ACCACCATGCCCAGCTAACTTTTGTGTTTTTTAGTACAGACAGGGTTTCGCCA|CA|AGAGTGGAAGAATTCACCCAACTCACAGGTAT
                                                      >chr2:10340140-10340440 - E=8e-16
TTGATGGTGCACTTCCATGGCTGGGATTGGCTTTAAAAAGGTCTTATCTCAAATTCCTTCCATGAAACAGAGTTTTTTTGAAGCCAACT
9
TAAAAGGCCTATGTAAAAATAATTATTCTTGCTGCACCTTATACAAATAATCTGGCCACAGCTGGGTGCGGTGGTTCACTTCTGTAATC

CCAGCACTTTGGGAGGCTGACATGGGTGGATCACCTGAGGTCAGGAGTTGGAGATCAGCCTGGCCAACATGGTGAGACCCCATCTCCA
```
